# Detection of clinical and neurological signs in apparently asymptomatic HTLV-1 infected carriers: Association with high proviral load

**DOI:** 10.1371/journal.pntd.0006967

**Published:** 2019-05-01

**Authors:** Michel E. Haziot, M. Rita Gascon, Tatiane Assone, Luiz Augusto M. Fonseca, Olinda do Carmo Luiz, Jerusa Smid, Arthur M. Paiva, Rosa Maria do N. Marcusso, A. C. Penalva de Oliveira, Jorge Casseb

**Affiliations:** 1 Institute of Infectious Diseases “Emilio Ribas” (IIER) of São Paulo, São Paulo, SP, Brazil; 2 Laboratory of Dermatology and Immunodeficiencies, Department of Dermatology, University of São Paulo Medical School, Brazil; 3 Institute of Tropical Medicine of São Paulo, São Paulo, SP, Brazil; 4 Department of Preventive Medicine, University of São Paulo Medical School, São Paulo, Brazil; University of Washington, UNITED STATES

## Abstract

Several studies suggest that HTLV-1 infection may be associated with a wider spectrum of neurologic manifestations that do not meet diagnostic criteria for HAM/TSP. These conditions may later progress to HAM/TSP or constitute an intermediate clinical form, between asymptomatic HTLV-1 carriers and those with full myelopathy. Our aim was to determine the prevalence of HTLV-1-associated disease in subjects without HAM/TSP, and the relationship between these findings with HTLV-1 proviral load (PVL). Methods: 175 HTLV-1-infected subjects were submitted to a careful neurological evaluation, during their regular follow up at the HTLV outpatient clinic of the Institute of Infectious Diseases “Emilio Ribas”, São Paulo city, Brazil. Clinical evaluation and blinded standardized neurological screening were performed for all the subjects by the same neurologist (MH). Results: After the neurological evaluation, 133 patients were classified as asymptomatic and 42 fulfilled the criteria for intermediate syndrome (IS). The mean age of the enrolled subjects was 46.3 years and 130 (74.3%) were females. Clinical classification shows that neurological symptoms (p<0.001), visual disorders (p = 0.001), oral conditions (p = 0.001), skin lesions (p<0.001), bladder disorders (p<0.001), and rheumatological symptoms (p = 0.001), were strongly associated to IS, except for disautonomy (p = 0.21). A multivariate analysis revealed that HTLV-1 proviral load, oral conditions, bladder disorders and rheumatological symptoms were independently associated with the IS. Conclusions: We found some early alterations in 42 patients (24%), particularly the presence of previously not acknowledged clinical and neurological symptoms, among subjects previously classified as "asymptomatic", who we reclassified as having an intermediate syndrome.

## Author summary

At least 5–10 million people live with the Human T-Cell Lymphotropic Virus type 1 (HTLV-1) worldwide, and around 0.25–5% of them may develop HTLV-1-associated myelopathy/Tropical spastic paraparesis (HAM/TSP), which is associated with chronic inflammation. In this study, involving 175 HTLV-1-infected subjects originally classified as asymptomatic, we found that 42 of them in reality presented some early clinical conditions, including alterations related not only to the neurological system, but also to the eyes and the skin. We called such conditions intermediate syndrome. Thus, it seems reasonable to suggest that all HTLV-1-infected subjects should be monitored for symptoms that may arise earlier in the course of their infection.

## Introduction

HTLV-1, a human retrovirus, is the causative agent of Adult T Leukemia/Lymphoma (ATLL) and HTLV-1-associated myelopathy (HAM/TSP)[1], at least 5–10 million people infected worldwide, almost 5–10% of them in Brazil [2]. However high, such numbers may be an underestimate since only 2/3 of the world has been mapped for HTLV infection [3]. Clinically, HAM/TSP is characterized by muscle weakness, hyperreflexia, spasticity in the lower extremities and urinary disturbances associated with preferential damage to the thoracic spinal cord [4]. HTLV-1 has been shown to be associated not only with HAM/TSP but also with several inflammatory diseases, such as alveolitis, polymyositis, arthritis, infective dermatitis, Sjögren syndrome and uveitis [5–9]. In addition, sensory and gait abnormalities, isolated bladder dysfunction, erectile dysfunction, and sicca syndrome, have all been reported among HTLV-1–infected individuals without HAM/TSP [10].

Several neurological manifestations that are not explained by myelopathy have been described in so-called symptomatic persons, such as peripheral polyneuropathy, myositis, dysautonomia and cognitive alterations, as well as cranial neuropathies, movement disorders and an amyotrophic lateral sclerosis (ALS)-like syndrome [11]. Despite the fact that few patients (<10%) will develop classical syndromes (ATLL and HAM/TSP), preliminary observations indicate that other symptoms and subclinical neurological disturbances can develop in those individuals [12], but they have not been well-defined as new clinical outcomes related to early inflammation process.

In addition, peripheral neuropathy is significantly more frequent in the seropositive group. In a study with 153 HTLV-1-infected carriers, the presence of higher frequency of motor and bladder dysfunctions in HTLV-1 patients as compared with uninfected control subjects was found [13]. Those data suggest that HTLV-1-infected individuals may exhibit a wide variety of neurological manifestations distinct from the classical picture of HAM/TSP [13]. It is unclear whether such manifestations share a common characteristic with the spinal cord disease.

This study aims to demonstrate that some clinical conditions, neurological finds and HTLV-1 proviral load may be associated with further development of full-blown HAM/TSP, in individuals considered free of the disease according to currently used criteria for its diagnosis. To do so we studied patients from a large cohort of asymptomatic HTLV-1 carriers who have been followed for more than twenty years.

## Methods

Our cohort has 659 HTLV-1-only infected subjects, with its inception dating back to 1997 [14]. It is an open cohort, with new patients added at a rate of approximately 50 per year. To avoid confounding, patients with HIV and/or HCV co-infections were excluded from this research, as well as other 90 patients who did not have a regular follow-up. For this study, 175 cases with confirmed HTLV-1 infection and without a HAM/TSP diagnosis were included and blindedly assessed during their regular follow-up at the HTLV outpatient clinic of the Institute of Infectious Diseases “Emilio Ribas”, Sao Paulo city, Brazil.

### Clinical evaluation

Clinical evaluation and a standardized screening neurological examination were performed by MH (a board-certified neurologist, and blinded for HTLV-1 clinical condition) for all subjects. Only symptoms/signals already associated with HTLV-I infection in previous reports were considered, and they should have no other clinical explanation. Each patient had at least one neurological/clinical evaluation, and a standardized questionnaire was used, with separate questions for clinical and neurological aspects [15].

### Case definition

HAM/TSP diagnostic criteria was based on recommendations from an international consortium [16]. Briefly, definite HAM/TSP is a non-remitting progressive spastic paraparesis with sufficiently impaired gait to be perceived by the patient. Sensory symptoms or signs may or may not be present but when present, they are subtle and without a clear-cut sensory level. Urinary and anal sphincter signs or symptoms may or may not be present, and the presence of anti-HTLV-1 antibodies in the cerebro spinal cord fluid (CSF).

Probable HAM/TSP was defined by a monosymptomatic presentation: spasticity or hyperreflexia in the lower limbs or isolated Babinski sign with or without subtle sensory signs or symptoms, or neurogenic bladder only confirmed by urodynamic tests. For both definite and probable definitions, clinicians must exclude an array of disorders that can mimic HAM/TSP.

We described a possible intermediate state of HAM not fulfilling the classical definition (16). To be considered as an intermediate syndrome case the patient must present more three signs, found during a neurological evaluation, with the investigator blinded to the patient’s HTLV status.

For this present study, only clinical findings previously associated with HTLV-1 were considered, such as dermatological, ophthalmological, rheumatological, urinary, disautonomic, and oral changes [17]. Neurological evaluation included tests of strength in upper and lower limbs, cranial nerves function and patellar, biceps and plantar reflexes, as well as an appraisal of the vibration sense. The presence of minimal changes in muscular strength or gait was explored: subjects were asked to walk on their heels, toes, tandem gait, and rise from a chair without help from their arms.

### Database

A large clinical and laboratory database has been organized on an internet based platform using REDCap, software developed at the Vanderbilt University by an informatics core. All clinical data, which have been updated on a regular basis over the last 20 years, were entered into a specific REDCap database [18].

### DNA HTLV-1 proviral load

HTLV-1 proviral load was quantified by real-time PCR, using primers and probes targeting the pol gene: SK110 and SK111, the internal HTLV-1 Taq Man probe was selected using Oligo (National Biosciences). All samples were run in duplicate, and results expressed as HTLV-1 DNA copies/10^4^ peripheral blood mononuclear cells (PBMCs), as described elsewhere [19].

### Ethical issues

The Ethical Board of the IIER approved the protocol (Number 86379218.6.1001.0061). We obtained signed informed consent from all participants prior to study inclusion, and all participants were adults.

### Statistical analysis

Statistical analysis was conducted using Student’s t-test for parametric data, and the chi-square test for proportions. Bivariate logistic analysis was performed to identify independent variables associated with the intermediate syndrome (IS). Variables associated with the outcome at a significance level of p<0.20 (IS) in the bivariate analysis were included in a multivariate logistic model, in a stepwise forward fashion. Such variables were: visual symptoms, skin lesions, oral conditions, bladder disfunction, and rheumatological conditions. The best fitting model was selected. The logistic analysis was performed with the aid of Stata 12 software (StataCorp. 2011. Stata: Release12. Statistical Software. College Station, TX).

## Results

We enrolled 175 HTLV-1 patients on this study and classified them as having or not criteria for the diagnosis of the intermediate syndrome. Based on a thorough neurological examination, 42 patients met the criteria for making the diagnosis of the intermediate syndrome, whereas 133 did not and were called “asymptomatic” (not having the intermediate syndrome). All of them had intermediate symptoms that were classified as probable HAM/TSP at entry, primarily neurogenic bladder confirmed by urodynamic study. Table 1 shows the univariate analyses of socio demographic variables and proviral load of all volunteers; mean age of the enrolled subjects (n = 175) was 46.3 years and 130 (74.3%) were females. Most of the patients were white (56.5%), and the PVL from the IS cases was six times that from patients without IS (p<0.001). Clinical classificafion on Table 2 shows that neurologic symptoms/signals (p<0.001), visual disorders (p = 0.001), oral manifestations (p = 0.001), skin lesions (p<0.001), bladder disorders (p<0.001), and rheumatologic symptoms (p = 0.001), were strongly associated to IS, except for disautonomy (p = 0.21).

**Table 1:** Univariate analyses of socio demographic variables and their association with the intermediate syndrome.

Variables	Total N(%)	Intermediate syndrome		OR (IC95%)	P value
		**No** **n (%)**	**Yes** **n (%)**		
**Gender**					
Male	45 (100)	32 (71.1)	13 (28.9)	1	
Female	130 (100)	101 (77.7)	29 (22.3)	0.70 (0.33–1.52)	0.374
**Age** Mean(SD)	46.3 (13.3)	46.7 (13.2)	44.1 (13.9)	0.98 (0.95–1.02)	0.400
Skin color**					
White	70 (100)	60 (85.7)	10 (14.3)	1	
Black*	49 (100)	39 (79.6)	10 (20.4)	1.54 (0.58–4.04)	0.382
Asian	05 (100)	04 (80.0)	01 (20.0)	1.50 (0.15–14.83)	0.729
DNA HTLV-1 Proviral Load*** Mean (SD)	60 (190)	25 (106)	169 (319)	1.004 (1.001–1.007)	0.007

*Black and brown

** Missing data: 51 subjects

***(copies/10^4^ PBMC)

**Table 2:** Conditions associated with HTLV-1 seropositivity among patients with and without the intermediate syndrome.

*Clinical Symptoms*	Total N(%)	Intermediate syndrome		OR (95%IC)	P value
		No N (%)	Yes N (%)		
**Disautonomy**	175 (100)	133 (100)	42 (100)		
Yes	07 (04)	04 (03)	03 (07)	2.4 (0.54–11.56)	0.247
No	168 (96)	129 (97)	39 (93)	1	
Neurological symptoms*	175 (100)	133 (100)	42 (100)		
Yes	56 (32)	14 (11)	42 (100)	-	-
No	119 (68)	119 (89)	0 (-)		
**Visual Symptoms**	175 (100)	133 (100)	42 (100)		
Yes	33 (18)	13 (10)	20 (48)	8.39 (3.65–19.30)	0.0001
No	144 (82)	120 (90)	22 (52)	1	
**Oral Manifestations**	175 (100)	133 (100)	42 (100)		
Yes	31 (18)	12 (09)	19 (45)	8.33 (3.56–19.47)	0.0001
*No*	144 (82)	121 (91)	23 (55)	1	
**Skin lesions**	175(100)	133 (100)	42 (100)		
Yes	60 (34)	30 (22)	30 (72)	8.58 (3.92–18.78)	0.0001
No	115 (66)	103 (77)	12 (28)	1	
**Bladder disorder**	175 (100)	133 (100)	42 (100)		
Yes	43 (25)	15 (11)	28 (66)	15.73 (6.81–36.33)	0.0001
No	132 (75)	118 (89)	14 (34)	1	
**Rheumatological symptoms**	175 (100)	133 (100)	42 (100)		
Yes	53 (30)	26 (20)	27 (64)	7.40 (3.45–15.88)	0.0001
No	122 (70)	107 (80)	15 (36)		

*Neurological signals/symptoms: Neurological evaluation included tests of strength in upper and lower limbs, cranial nerves function and patellar, biceps and plantar reflexes, as well as an appraisal of the vibration sense. The presence of minimal changes in muscular strength or gait, and the subjects were asked to walk on their heels, toes, tandem gait, and rise from a chair without help from their arms.

On a multivariate model analysis, including gender, age, and PVL and several clinical conditions, such as oral conditions, bladder disorders and rheumatological symptoms were independently associated with SI outcome. In this same model, all these variables and age were also significantly associated with the outcome, when included as continuous variables (Table 3).

**Table 3:** Logistic regression of variables associated with intermediate syndrome.

*Clinical Symptoms*	OR* (95%IC)	P value	OR** (95%IC)	P value
**Age**	0.98 (0.95–1.02)	0.400*	0.87 (0.79–0.97)	0.013***
**Gender**				
Females	0.71 (0.33–1.52)	0.374	0.52 (0.09–2.97)	0.462
Males	1		1	
**DNA HTLV-1 Proviral Load**	1.004 (1.001–1.007)	0.007*	1.04 (1.01–1.06)	0.007***
**Oral Conditions**				
Yes	8.33 (3.56–19.47)	0.0001	84.41 (5.43–1311.09)	0.002
*No*	1		1	
**Bladder disorders**				
Yes	15.73 (6.81–36.33)	0.0001	173.15 (9.37–3199.06)	0.001
No	1		1	
**Rheumatological symptoms**				
Yes	7.40 (3.45–15.88)	0.0001	125.13 (6.89–2272.34)	0.001
No			1	

*Crude OR

**adjusted OR

***p value for trend

Table 4 shows that the presence of more than three or more signs and/or symptoms was significantly associated with the intermediate syndrome (p = 0.006), therefore the the cut-off point for that diagnosis was set at this point.

**Table 4:** Association of the neurologic symptoms with HTLV-1 DNA proviral load.

Numbers of symptoms	N. Cases	HTLV-1 Proviral Load Mean (±SD)	p value*
≤3	137	41 (±166)	0.006
>3	38	129 (±252)	

* teste t

Note: The signs and symptoms included were disautonomy, neurological and visual disorders, oral conditions, skin lesions, bladder disorders, and rheumatological symptoms.

## Discussion

This study aimed to define the early neurological disorders that can be present in HTLV-1-infected subjects. We found 24% of HTLV-1-infected patients from our outpatient service who were initially considered asymptomatic to have enough signs and symptoms putting them on a novel category, called intermediate syndrome. The correlation between some of their symptoms and the proviral load also reinforces the importance of such mild forms, which may constitute either an independent clinical intermediate syndrome or markers for an early diagnosis of HAM/TSP. A significantly PVL was also present in patients presenting three or more symptoms or signs.

HAM/TSP is a chronic progressive myelopathy characterized by bilateral pyramidal tract involvement with sphincter disturbances. Why only a small proportion of HTLV-1-infected individuals develops classical HAM/TSP is not known [11]. The main neurological symptoms of the disease are progressive and lead to deterioration in the quality of life, but minor neurological symptoms can also be found among HTLV-1 carriers [11]. In a study with 153 HTLV-1-infected carriers and 388 HTLV-2-infected subjects, the presence of neurological abnormalities was prospectively ascertained, with a higher frequency of motor and bladder dysfunctions in HTLV-1 as compared with uninfected control subjects [10]. All those data suggest that HTLV-1-infected individuals can exhibit a wide variety of neurological manifestations distinct from the classical picture of HAM/TSP [11]. It is unclear whether those manifestations share a common characteristic with this diagnosis.

We and others have shown a correlation between proviral load and Tax gene expression with the presence of HAM/TSP [14,19,20]. We hypothesize that the burden of HTLV-1 was correlated with neurological disturbances that fall short of HAM/TSP, and with cognitive dysfunction. Demonstration of that hypothesis might provide a link between HTLV-1 burden and early neurological dysfunctions, providing impetus to the development of methods aiming to reduce the proviral load in infected subjects.

Perhaps the explanation for the observed neurological findings lies in the white matter. The white matter present in the CNS has the important function of transporting neural signals from subcortical regions to the cortex and from the cortex to the subcortical regions. In the CNS, ischemic and traumatic injuries often result in significant functional deficit, most of which can be attributed to white matter dysfunction [21–23]. In the case of HTLV-1, there is participation of components of the inflammatory response on the mechanisms underlying the demyelination process characteristic of this disease [20].

We found that HTLV-1 infection is associated with a variety of clinical manifestations occurring in patients who either do not have or who did not have developed full HAM/TSP yet. The correlation between some of their symptoms and the proviral load also reinforces the importance of such milder forms, which may constitute either an independent clinical SI or markers for an early diagnosis of HAM/TSP. A significantly higher proviral load was present in patients presenting more than three symptoms/signs, a cut-off point that can constitute a surrogate marker for clinical progression.

In conclusion, this preliminary report identified the presence of some clinical and neurological symptoms, in subjects classified originally as "asymptomatic," which may be promising markers for early HAM/TSP progression. This knowledge may contribute for a stricter clinical vigilance of the so-called asymptomatic HTLV-1 positive patients, prompting the introduction of a treatment for the infection, if and when it becomes available in the future.

## Supporting information

S1 Checklist STROBE checklist. (DOC)
