# Supplementary material for: Detection of clinical and neurological signs in apparently asymptomatic HTLV-1 infected carriers: Association with high proviral load
Source: PLoS Negl Trop Dis. 2019 May 1;13(5):e0006967. doi: 10.1371/journal.pntd.0006967 (PMC6513103; doi:10.1371/journal.pntd.0006967)
Supplement: S1 Checklist — (DOC) [file pntd.0006967.s001.doc]

STROBE Statement—Checklist of items that should be included in reports of ***cross-sectional studies***

|  | Item No | Recommendation |
| --- | --- | --- |
| **Title and abstract** | 1 | (*a*) Detection of clinical and neurological signs in apparently asymptomatic HTLV-1 infected carriers: association with high proviral load. |
|  |  | (b) The aim was to determine the prevalence of HTLV-1-associated disease in subjects without HAM/TSP, and the relationship between these findings with HTLV-1 PVL Methods: 175 HTLV-1-infected subjects were submitted to to a careful neurologic evaluation, during their regular follow up at the HTLV outpatient clinic of the Institute of Infectious Diseases “Emilio Ribas”, São Paulo city, Brazil. Clinical evaluation and blinded standardized neurological screening were performed for all the subjects by the same neurologist (MH). Results: After the neurologic evaluation, 133 were classified asymptomatic and 42 patients fulfilled the criteria for intermediate syndrome (IS). The mean age of the enrolled subjects was 46.3 years and 130 (74.3%) were females. Clinical classificafion shows that neurological symptoms (p<0.001), visual disorders (p=0.001), oral conditions (p=0.001), skin lesions (p<0.001), bladder disorders (p<0.001), and rheumatologic symptoms (p=0.001), were strongly associated to IS, except for disautonomy (p=0.21). The multivariate analysis revealed that viral load, oral conditions, bladder disorders and rheumatologic symptoms were independently associated with the IS. Conclusions: Some early alterations in the neurocognitive sphere, as well as the presence of other clinical and neurologic symptoms in 42 subjects (24%) previously classified as "asymptomatic", who were then reclassified as having an intermediate syndrome. |
| Introduction | | |
| Background/rationale | 2 | The causative role of HTLV-1 infection is yet to be proven for most of those observations, which were mainly based on small case series. Several neurological manifestations that are not explained by myelopathy have been described in so-called asymptomatic persons, such as peripheral polyneuropathy, myositis, dysautonomia and cognitive alterations; as well as cranial neuropathies, movement disorders and an amyotrophic lateral sclerosis (ALS)-like syndrome have also been reported. Despite the fact that few patients (<5%) will develop classical syndromes (ATLL and HAM/TSP), preliminary observations indicated that other symptoms and subclinical neurological disturbances can develop in those individuals.  In addition, peripheral neuropathy was significantly more frequent in the seropositive group. In a study with 153 HTLV-1-infected carriers, the presence of neurologic abnormalities was prospectively ascertained and higher frequency of motor and bladder dysfunctions in HTLV-1 patients as compared with uninfected control subjects. All those data suggested that HTLV-1-infected individuals may exhibit a wide variety of neurological manifestations distinct from the classical picture of HAM/TSP. It is unclear whether those manifestations share a common characteristic with this diagnosis. |
| Objectives | 3 | This study aims to demonstrate the relationship between clinical manifestations, neurological finds and HTLV-1 proviral load to be associated with HAM/TSP, in individuals without myelopathy, according to currently used criteria for HAM/TSP diagnosis, from a large cohort of asymptomatic HTLV-1 carriers. Such symptoms and conditions may later progress to HAM/TSP itself (ie. pre-clinical myelopathy symptoms) intermediate between the asymptomatic carriers and those with myelopathy. |
| Methods | | |
| Study design | 4 | A cross-sectional involving HTLV-1 subjects |
| Setting | 5 | HTLV outpatient clinic from Institute of Infectious Diseases Emilio Ribas” ,Sao Paulo, Brazil |
| Participants | 6 | (a) followed up 659 HTLV-1-only infected subjects since 1997; of these, 175 HTLV-1 patients were included in this study from August 2016 to March 2018. |
| Variables | 7 | age, gender, ethnicity, DNA HTLV-1 proviral load , and clinical symptoms (disautonomy, neurological symptoms, visual symptoms, oral manifestations, skin lesions, bladder disorders) |
| Data sources/ measurement | 8* | For each variable of interest, give sources of data and details of methods of assessment (measurement). Describe comparability of assessment methods if there is more than one group |
| Bias | 9 | The same researcher evaluated all subjects |
| Study size | 10 | 175 HTLV-1 patients were included in this study from August 2016 to March 2018. |
| Quantitative variables | 11 | HTLV-1 DNA proviral load |
| Statistical methods | 12 | (a) Statistical analysis was conducted using Student’s t-test for parametric data, and the chi-square test for proportions. Bivariate logistic analysis was performed to identify independent variables associated with intermediate syndrome (IS). |
|  |  | (b) Variables associated with the outcome at a significance level of p<0.20 (IS) in the bivariate analysis were included in a multivariate logistic model. Logistic analysis was performed with the aid of Stata 10 software (StataCorp. 2009. Stata: Release 10. Statistical Software. College Station, TX). |
|  |  | (c) |
|  |  | (*d*) If applicable, describe analytical methods taking account of sampling strategy |
|  |  | (*e*) IC 95% ; p≤0.05 |
| Results | | |
| Participants | 13* | (a) We enrolled 175 HTLV-1 patients on this study and classified them as having or not criteria for the diagnosis of the intermediate syndrome. Based on a thorough neurological examination, 42 patients met the criteria for making the diagnosis of the intermediate syndrome whereas 133 did not, and were called “asymptomatic” (not having the intermediate syndrome). |
|  |  |  |
|  |  |  |
| Descriptive data | 14* | (a) The univariate analyses of socio demographic variables and proviral load of all volunteers; mean age of the enrolled subjects (n=175) was 46.3 years and 130 (74.3%) were females. Most of the patients were white (56.5%), and the DNA HTLV-1 proviral load (PVL) from the IS cases was six times that from patients without IS (p<0.001). |
|  |  |  |
| Outcome data | 15* | Clinical classification shows that neurologic symptoms (p<0.001), visual disorders (p=0.001), oral manifestations (p=0.001), skin lesions (p<0.001), bladder disorders (p<0.001), and rheumatologic symptoms (p=0.001), were strongly associated to IS, except for disautonomy (p=0.21). |
| Main results | 16 | (a) On a multivariate model analysis, including gender, age, and HTLV-1 DNA proviral load and several clinical conditions, such as viral load, oral conditions, bladder disorders and rheumatologic symptoms were independently associated with SI outcome. In this same model all these variables and age were also significantly associated with the outcome, when included as continuous variables. |
|  |  | (*b*) Report category boundaries when continuous variables were categorized |
|  |  | (*c*) If relevant, consider translating estimates of relative risk into absolute risk for a meaningful time period |
| Other analyses | 17 | Report other analyses done—eg analyses of subgroups and interactions, and sensitivity analyses |
| Discussion | | |
| Key results | 18 | We found 24% of HTLV-1-infected patients from our outpatient service who were initially considered asymptomatic to have enough signs and symptoms putting them at a novel category, called intermediate syndrome. They present various clinical manifestations occurring in patients who either do not have or who did not have develop HAM/TSP yet. The correlation between some of their symptoms and the proviral load also reinforces the importance of such mild forms, which may constitute either an independent clinical intermediate syndrome (IS) or markers for an early diagnosis of HAM/TSP. A significantly higher proviral load (PVL) was also present in patients presenting three or more symptoms or signs. |
| Limitations | 19 | The main limitation of this study was the sample size, because it can influence on outcome. |
| Interpretation | 20 | We found that HTLV-1 infection is associated with a variety of clinical manifestations occurring in patients who either do not have or who did not have develop full HAM/TSP yet. The correlation between some of their symptoms and the proviral load also reinforces the importance of such milder forms, which may constitute either an independent clinical SI or markers for an early diagnosis of HAM/TSP. A significantly higher proviral load was present in patients presenting more than three symptoms/signs, a cut-off point that can constitute a surrogate marker for clinical progression. |
| Generalisability | 21 | This preliminary report identified the presence of clinical and neurologic symptoms, in subjects classified originally as "asymptomatic," which may be promising markers for early HAM/TSP progression. Altogether, new approaches generate insights, potentially affecting our understanding of the natural history of the neurologic disease and guiding new clinical endpoints, the larger impact being the characterization of a new clinical entity related to HTLV-1-infected subjects. Thus, the data generated on this research may prove useful to evaluate what is the best approach to identify surrogate markers for the early symptoms of HAM/TSP (intermediate syndrome). |
| Other information | | |
| Funding | 22 | Support: Fapesp: 2016/03025-2; |

*Give information separately for exposed and unexposed groups.

**Note:** An Explanation and Elaboration article discusses each checklist item and gives methodological background and published examples of transparent reporting. The STROBE checklist is best used in conjunction with this article (freely available on the Web sites of PLoS Medicine at http://www.plosmedicine.org/, Annals of Internal Medicine at http://www.annals.org/, and Epidemiology at http://www.epidem.com/). Information on the STROBE Initiative is available at www.strobe-statement.org.
